# Unexpected Significantly Reduced FDG Uptake in the Cerebrum Compared Against Normal Liver Uptake in a Dying Patient

**DOI:** 10.4274/Mirt.136

**Published:** 2012-08-01

**Authors:** Luke I. Sonoda, Bal Sanghera, Gill Vivian, Wai Lup Wong

**Affiliations:** 1 Mount Vernon Hospital, Paul Strickland Scanner Centre, Middlesex, United Kingdom

**Keywords:** Fluorodeoxyglucose F18, glucose metabolism disorders, brain vascular disorders, liver, cardiac muscle, physiological processes, fever of unknown origin

## Abstract

F-18 FDG PET-CT scanning plays an important role in the management of fever of unknown origin (FUO). Some elderly patients with FUO can be in their terminal stage of life. An elderly woman was referred for a PET-CT scan to find the etiology of FUO. The scan was inconclusive but showed significantly reduced FDG uptake in the brain and heart, despite normal physiological uptake in the liver and bowel. The patient deceased within the hour post scan. Contrary to common belief, we have shown that cerebral glucose metabolism via cerebral perfusion may be compromised before hepatic and bowel perfusion in a dying patient.

**Conflict of interest:**None declared.

## INTRODUCTION

Although F-18 FDG PET-CT scanning has now become widely available as a new radiological and nuclear medicine investigation, it is by large considered as a tool of non-acute clinical management such as oncology staging (1), monitoring response to therapies (2), detection of disease recurrence (3) and detection of unknown primary malignancies (4) or infection/inflammation (5). Hence, it is quite unusual to encounter a PET-CT scan of a patient who slowly deteriorated to pass away within 45 minutes after the scan. Unexpectedly we performed a PET-CT scan of a dying patient that demonstrated some unexpected cerebral FDG uptake pattern compared to other remaining organs such as heart, liver and gastrointestinal tract.

## CASE REPORT

**Figure 1:**
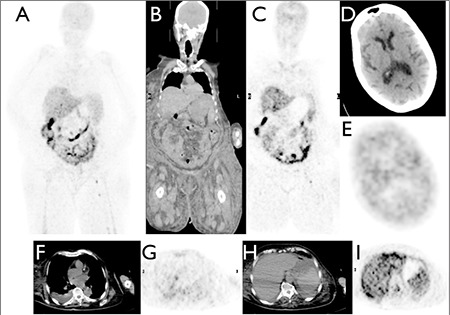
FDG PET-CT scan of the patient. A: MIP PET, B and C: coronal CT and PET, D and E: axial CT and PET at the level of the brain, F and G: axial CT and PET at the level of the heart, H and I: axial CT and PET at the level of the liver

A 70 year-old female, with a reduced level of consciousness but who was stable and capable to talk slowly and communicate at the time of investigation, was referred for a F-18 FDG-PET-CT scan to investigate FUO (6,7,8). The PET-CT scan demonstrated moderate subcutaneous oedema (Figure 1 A, B and C) and moderate right pleural effusion (Figure 1 F and G) due to fluid overload, but no cause of FUO was identified.

Reduced cardiac FDG uptake (SUV max=1.55, SUV mean=0.95) was considered as a result of ‘fasting’ (9), with no feeding for 6 hours prior to the scan (Figure 1 G). The serum glucose level was 5.6 mmol/l. 

The patient had no relevant past medical history and was not on any medication. In particular, the patient did not suffer from any forms of dementia nor pathology involving cerebrum such as Creutzfeldt-Jakobs disease. Physical examinations and routine blood tests were all normal and previous imaging investigations including CT, MRI and abdominal ultrasound did not find significant abnormalities.

Diffusely decreased cerebral and cerebellar FDG uptake (SUV max=2.47, SUV mean=1.94) was an unexpected finding (Figure 1 D and E), especially because hepatic FDG uptake and intestinal FDG uptake appeared normal (Figure 1 H and I) (10).

The patient deteriorated unexpectedly and was deceased 45 minutes after the scan despite attempts of resuscitation. Autopsy did not reveal significant pathology including normal appearances of the cerebrum.

**Figure 2:**
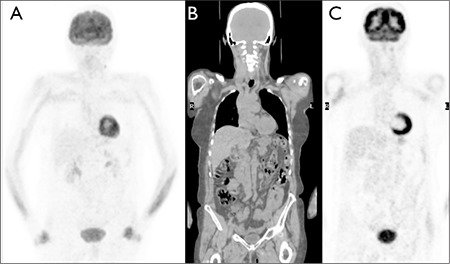
Example of a normal FDG PET-CT scan. A: MIP PET, B and C: coronal CT and PET

Figure 2 is an example of a normal F-18 FDG PET-CT scan for reference. Normal physiological FDG uptake was seen in the brain (SUV max=12.9, SUV mean=6.5), heart, liver and bowel (11,12).

## LITERATURE REVIEW AND DISCUSSION

It is widely believed cerebral perfusion and glucose metabolism may be sustained until all peripheral perfusion is compromised in a dying patient. However, this case unexpectedly demonstrated that cerebral perfusion and glucose metabolism appeared compromised before hepatic and intestinal perfusion and glucose metabolism.

There exist many physiological mechanisms, such as peripheral vascular shutdown in the event of large blood volume loss, to protect central blood circulation (but there is no such vasoconstriction mechanism within the cerebral vascular system itself) (13). But in large, it appears to be a common belief that the cerebral perfusion and glucose metabolism would be the last to be compromised. This myth has been believed and indirectly taught in many medical schools. In this case report, we would like to highlight that this myth may not always be the case.

Diffuse decrease in intracranial glucose metabolism may occur in various conditions (14). Organic diseases such as Alzheimer’s dementia and amyloid deposition (15), intracranial infection (16), intracranial atrophy caused by long-standing toxic substances such as alcohol (17), systemic metabolic diseases such as diabetes and resistance to insulin (18) are the common known causes of diffuse decrease in glucose metabolism in the brain. In this case report, the patient did not suffer from any of these diseases and was not on any medications except for an appropriate level of intravenous saline infusion.

A potential learning point of this case report is when significantly reduced cerebral FDG uptake was observed in a PET-CT scan, then staff immediately need to ascertain if the study was conducted appropriately e.g. injection extravasation or wrong dose injection. After exclusion of such operational errors, these abnormal findings should be discussed with duty doctors immediately and appropriate clinical management/resuscitation should be initiated (19).
